# Irritable Bowel Syndrome and Depression: A Shared Pathogenesis

**DOI:** 10.7759/cureus.3178

**Published:** 2018-08-21

**Authors:** Tatenda A Mudyanadzo, Chandanbindya Hauzaree, Oksana Yerokhina, Nalini Narayanan Architha, Hasan M Ashqar

**Affiliations:** 1 Surgery Research, University of South Alabama, Mobile, USA; 2 Pathology, SSR Medical College, Phoenix, MUS; 3 Internal Medicine, CiBNP, Fairfield, CA, USA; 4 Medicine, CiBNP, Fairfield, CA, USA; 5 Medicine, CIBPN, Fairfield, CA, USA

**Keywords:** irritable bowel syndrome depression, depression ibs cytokines, gut brain axis ibs, gut brain axis depression, rome iv, immune system ibs depression, hypothalamic pituitary adrenal axis depression, pathophysiology ibs depression

## Abstract

It is common knowledge that dysfunction of the immune and neuroendocrine systems, in addition to neuroplasticity, is among the pathways that underlie irritable bowel syndrome (IBS) and depression. From as early as the 1950s, the association of IBS with psychiatric disease was postulated; however, the exact mechanism remains elusive. There has been considerable research into the association of IBS and depression over the last years; research into the gut-brain axis and alterations in gut microbes have gained momentum to spell out the relationship between depression and IBS. Evidence from these researchers indicate the dysfunction of homeostatic coping mechanisms; corticotropin-releasing factor appears to be at the core of this dysfunction. The multifactorial etiology of both depression and IBS hinders a universal, one-strategy-fits-all treatment approach to patients with comorbid depression and IBS.

This review analyzes the pathophysiology that associates these two conditions; it explores the bidirectional communication between the brain and the gastrointestinal tract, and how these influence the endocrine and immune systems.

Review articles, clinical trials and randomized controlled trials that analyzed the association of depression and IBS were identified by searching PubMed, Google Scholar, and articles in PMC databases. Full texts written in English and available via these search engines were selected for the synthesis of this review.

Alterations to the gut-brain axis, intestinal microbiota, and the neuro-immune system may be the cornerstone to the association of IBS and depression. This literature review opens alternate therapeutic approaches to comorbid IBS and depression and encourages further research into this topic.

## Introduction and background

“All disease begins in the gut,” - Hippocrates

A 32-year-old woman reported marked flatulence, chronic abdominal pain relieved with defecation, an exacerbation of abdominal pain, intermittent constipation lasting greater than three days at a time, and depression symptoms. Physical examination revealed an abdomen with borborygmi, and she burped with abdominal palpation. Examination of the stool revealed semi-formed fecal material. Irritable bowel syndrome (IBS) and depression were diagnosed. What is the relationship between these two conditions?

Definitions and estimates of prevalence

IBS is a common gastrointestinal tract (GIT) disorder, with a female to male predominance of 2:1 [1-3] and a prevalence estimated at 12% [4,5]. The hallmark of IBS is the periodic abdominal pain in the absence of structural or biochemical abnormalities; the standard to meet a diagnosis of IBS is set by the Rome IV criteria [5-8]. Irritable bowel syndrome diagnostic criteria as defined by Rome IV requires a minimum of three months of periodic abdominal pain with at least one day per week of pain plus any two or more of the following symptoms: pain related to defecation; change in form/consistency of stool and reduced frequency of stool (fewer than three bowel movements per week).

IBS falls into a class of disorders termed functional gastrointestinal disorders (FGIDS) [6]; the other members in the group are functional abdominal bloating/distension, functional defecation disorder, and functional constipation [4-6].

A lot is known about the interrelation of FGIDS and mood disorders [9,10]; the co-occurrence of FGIDS and depression is estimated at 30% [9], thus, the moniker disorders of gut-brain interaction. Depression is a common mood disorder; it is estimated that 16.2 million adults in the United States had at least one major depressive episode in 2016. That translates to approximately 6.7% of all US adults [11]. Globally, the World Health Organization estimates that in 2013, 350 million individuals were affected by depression [12].

In this article, depression will be taken to include major depressive disorder (MDD) and persistent depressive mood which are two conditions with separate criteria in the Diagnostic and Statistical Manual of Mental Disorders, fifth edition (DSM-5).

MDD is a psychiatric disorder that is characterized by a minimum of two weeks of persistently low mood and/or loss of interest in previously pleasurable activities plus four or more of the following symptoms: low self-esteem, guilt, sleep disturbances, low energy, changes in appetite, poor concentration, and pain of unclear etiology [13,14].

Persistent depressive mood, also known as dysthymia, is a chronically subdued mood that lasts for at least two years [14]. Traditionally, depression has been attributed to an imbalance in the neurotransmitters serotonin, norepinephrine and dopamine in the brain, thus our approach to treating it with selective serotonin reuptake inhibitors (SSRIs) and serotonin-norepinephrine reuptake inhibitors (SNRIs) as primary care first-line therapies.

## Review

**Figure 1:**
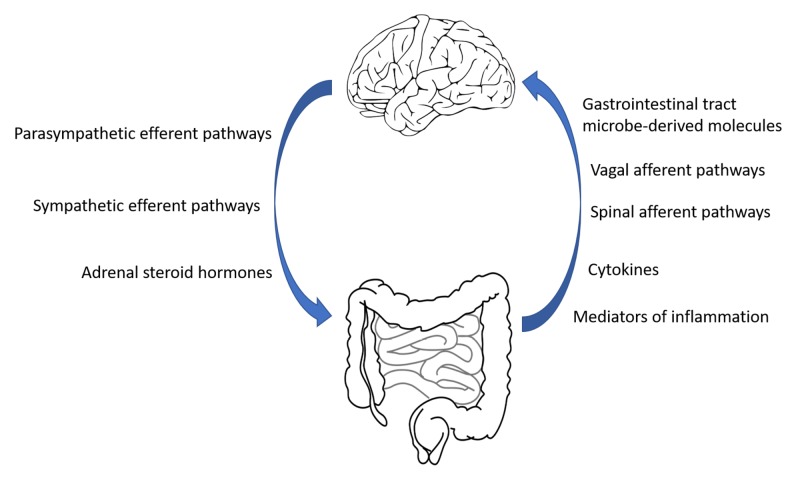
Gut-brain communication.

This literature review assesses the available data that relates to the link between IBS and depression; it will explore the central and peripheral mechanisms termed the gut-brain axis. A dysfunction of the gut-brain axis along with genetic factors, food sensitivity or allergies and infections are postulated as part of the pathophysiology of IBS and/or depression [2,15]. It is known that cytokines and inflammatory markers such as interleukin (IL)-6 and IL-10 [5,16] are important modulators of the immune system; these mediators of inflammation are at the forefront of intestinal inflammation and lead to conditions such as IBS. This inflammation alters gut microbes and the permeability of the gut mucosa. GIT microbes can produce most of the neurotransmitters found in the human brain [5,17]; along with low-grade inflammation, there is enhanced translocation of the produced neurotransmitters leading to activation of the hypothalamic-pituitary-adrenal (HPA) axis which is intrinsic to the pathophysiology of depression [5,18] and its related comorbidities. The bidirectional communication between the brain and the gut mucosa employs multiple information modalities as depicted in Figure 1 [5,19].

Some of the neurotransmitters from the nervous system are released by the neuroendocrine system, such as the adrenal medulla-produced dopamine. Similarly, some of the information from the GIT involved in bidirectional communication includes microbe-derived short-chain fatty acids such as propionic acid in addition to GIT hormones such as serotonin [5,19].

To guide us in the literature review, we required answers to the following two questions: one, is there a link between IBS and depression with regards to etiology and pathophysiology; and two, can we alter our approach to treating these conditions by evaluating the relationship between these two conditions.

To study the association between depression and IBS, a comprehensive search of PubMed, Google Scholar, and PMC was performed.

The determination to use these search engines was their ease of access, their frequent citation as well as use in health-related articles and their validity as a resource for obtaining information relating to this literature review. There were no ethical considerations while carrying out this literature search and the composition of the review.

**Figure 2:**
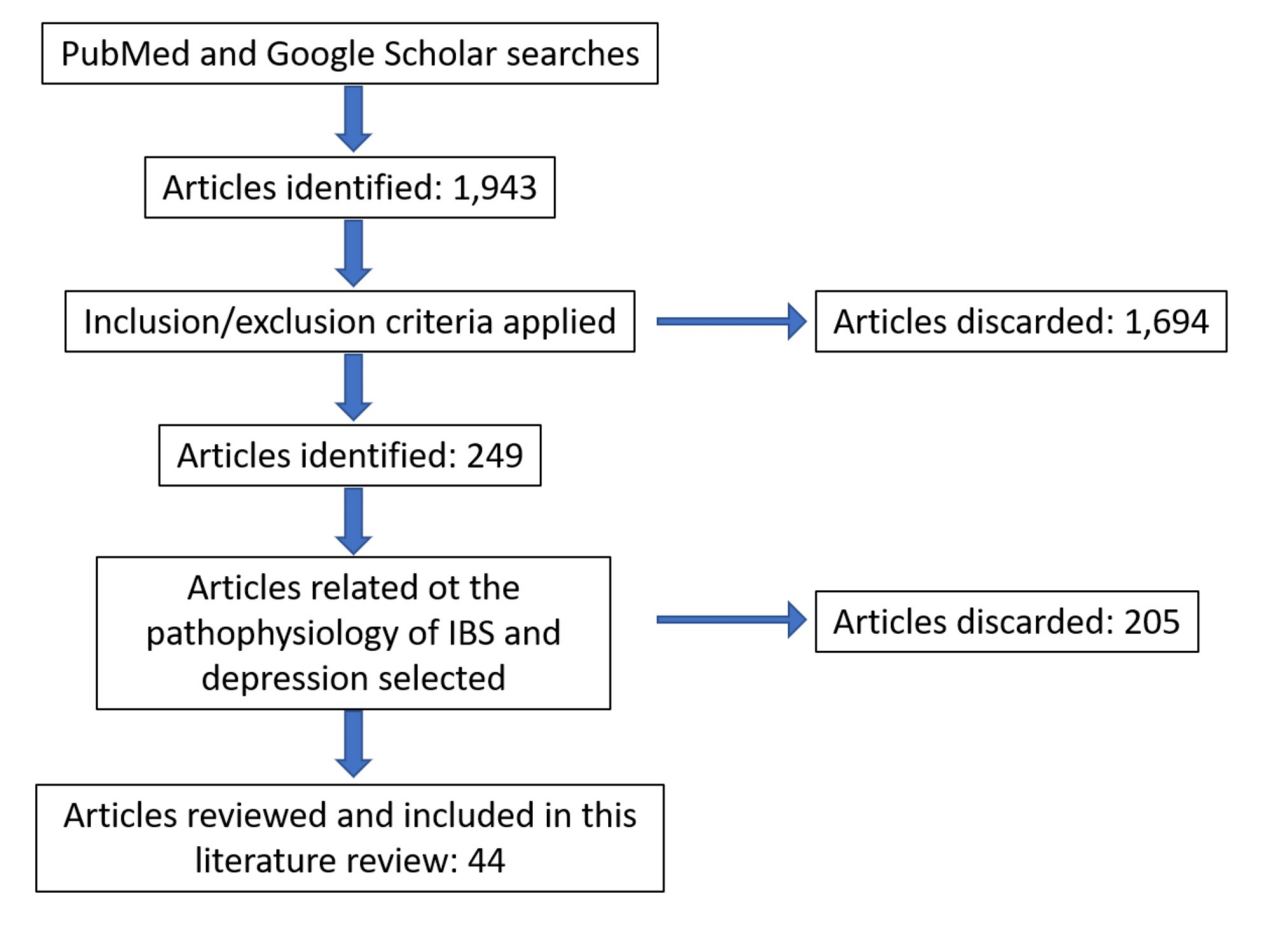
Data collection flow process. IBS: Irritable bowel syndrome

To streamline the search, the following keyword sets were utilized to search the databases: irritable bowel syndrome depression; depression IBS cytokines; gut-brain axis, IBS, depression; Rome IV; immune system, IBS depression; HPA, depression; pathophysiology IBS, depression. To narrow down and sift through the search outcomes, inclusion-exclusion criteria were applied. Inclusion criteria were that articles were less than five years old, related to humans, were clinical trials or literature reviews. In addition, articles whose titles overtly mentioned depression and irritable bowel syndrome were included. The titles of the remaining articles were reviewed, and only those with a title that matched the study of interest were included. Full texts were preferred over articles with abstracts only. The subject of this paper revolves around humans, and it was deemed necessary to utilize recent information that is in keeping with technology advances; thus the criterion to use articles regarding humans that were less than five years old. A study of the abstracts of the chosen articles was performed, and those with information deemed suitable for the review were shortlisted. English-only literature articles were reviewed to limit loss in translation. The references within the chosen articles were analyzed to assess the points of interest, and those references found to be relevant were assessed then included even if they were greater than five years old. The extracted data were analyzed for the synthesis of this review article. The data collection followed the flow process depicted in Figure 2.

There were a number of articles related to this topic, although only a handful of clinical trials came up and very few articles comprehensively discussed the treatment as postulated by the gut-brain axis. In spite of the fact that there was this limitation, the review did combine the selected articles and managed to highlight the association between IBS and depression.

The development of IBS is often preceded by a stressor to the patient; the stressor can be depression or any physical ailment [20,21]. The converse is true for depression where IBS can herald depression. This relation of reciprocity between IBS and depression needs further clarity to find the pathophysiology behind the two diseases as this will open alternate perspectives at evaluative, preventive and curative approaches to IBS and depression.

The nervous system plays an important role in homeostasis by monitoring and coordinating the function of different systems in the body; its action through the autonomic nervous system and neuroendocrine factors regulate the function of the GIT (Figure 1). The ability of the nervous system to transform its function, structure, and connections [20] in response to stressors is termed neuroplasticity. Any psychosocial disturbances can impact on nervous system neuroplasticity and this, in turn, will adversely affect downstream systems including the GIT.

Endocrine pathophysiology in IBS and depression

Corticotropin-Releasing-Factor (CRF)

The HPA axis is an influential pathway that can affect the GIT [22] through its action mediated by CRF [22,23]. CRF is a neuropeptide hormone produced by the paraventricular nucleus (PVN) of the hypothalamus in response to stress; elevated levels of CRF are linked with MDD and Alzheimer’s disease [24]. Some of the CRF receptors in the PVN of the hypothalamus, amygdala and locus coeruleus are involved in stress, affective, and pain circuits. Stimulation of these pathways could enhance pain and aversive behaviors of patients with IBS [3]. CRF enhances inflammation; at the same time through cortisol, it will cause immunosuppression [25]. Furthermore, circulating glucocorticoids enhance production of additional non-neuronal catecholamines to add to the stress hormones, thereby fueling additional stress and inflammation [22].

In the adult brain, neurogenesis in the hippocampus is related to a variety of cognitive functions including recall and learning [26]. Chronic stress and inflammation results in reduced neurogenesis within the hippocampus leading to atrophy and depressive behavior such as poor memory [26]. The presence of chronic inflammation and imbalance in inflammatory cytokines such as IL-6 and IL-10 will influence nervous system neuroplasticity to induce behavioral changes, cognitive impairment, and depression. At the same time, elevated levels of steroid hormones in stress favor an inflammatory environment that enhances an inflammatory cascade as alluded to above [22].

Modulation and Effect of GIT Hormones

Within the GIT, microbes express microbe-associated molecular patterns (MAMPS) and pathogen-associated molecular patterns that affect the sensory nervous system, and both the GIT endocrine and/or immune systems. Some of these MAMPS are exemplified by short-chain fatty acids (SCFAs) derived from indigestible matter that is degraded by microbes. Within the GIT, SCFAs modulate the function of macrophages, polymorphonuclear cells, and antigen-presenting cells; they enhance the integrity of the GIT epithelial barrier [22,27]. Distantly, the SCFAs play a restorative role for the blood-brain barrier and act as a messenger pathway to the brain microglia [28,29].

Apart from their effect on the immune system, SCFAs stimulate the release of GIT hormones such as glucagon-like peptide-1 (GLP-1), glucagon-like peptide-2 (GLP-2) and peptide YY (PYY). Through this pathway, GIT microbes exert a distant effect on multiple systems.

GIT Microbes

Changes in the composition of GIT microbes have been reported in IBS [2,3,30], and such changes can have far-reaching effects on the immune system and the enteroendocrine system. This manipulation of the gut microbe flora has been explored, and a fair amount of studies found an improvement in symptoms of IBS by using probiotics [3,30,31]; it is postulated that this approach will reduce proinflammatory markers, down-regulate T-cells and maintain the virtue of the intestinal barrier [3,31]. However, some studies did not show an improvement in the symptoms of IBS with the use of multispecies probiotics [30,32].

The contradictory information exemplified by the content of these studies [3,30-32] regarding the use of probiotics to alleviate depression among patients with IBS points to the need for further studies with vanguard technologies to further elucidate this approach to the treatment of depression among patients with IBS. All studies agree that there is a difference in GIT flora between healthy individuals and those with IBS [3,30-32].

Role of the Autonomic Nervous System

Sympathetic and parasympathetic innervation to the GIT is involved in maintaining homeostasis. The parasympathetic arm of the nervous system regulates smooth muscle activity and the secretory functions of the GIT; at the same time, it sends afferent neurons through the vagus nerve with non-nociceptive information to corticolimbic areas of the brain [2,3]. For example, it relays the gastrocolic reflex. Nociceptive information is transmitted by the sympathetic arm of the nervous system to terminate in the thalamus and sensory cortex. Efferent sympathetic nerves inhibit secretory and motor activities of the gut. These central autonomic regions interconnect with the hypothalamus, hippocampal areas, prefrontal cortex, and amygdala which are areas that regulate emotion and cognitive behavior and hence are related to depression.

Altered bowel movements and abdominal pain are the cornerstones in the diagnosis of IBS, and the function of the nervous system is related to these symptoms [3,33]. The loss of balance between the sympathetic-parasympathetic systems has been found to be present in IBS patients when compared to healthy controls [3]. This was supported by a study of female patients with IBS with marked constipation; it was found that they had lower vagal activity compared to matched controls [3]. The fight or flight effects of the sympathetic nervous system enhanced by CRF additionally increase constipation [3,33].

Indeed, IBS, as it occurs with depression, are both associated with dysfunction of the autonomic nervous system.

Relationship of pain with IBS and depression

Pain Hypersensitivity

Increased pain sensitivity among patients with IBS has been comprehensively demonstrated for quite a while and is considered one of the markers of IBS [2,3,34]. Exaggerated pain in response to stimulation of the rectum was initially noted by Ritchie in 1972 [2,3]. It has been noted that some patients with IBS are hypervigilant, have pain anticipation and experience emotional hypersensitivity in interpersonal interaction [3].

Post-Infectious IBS

Several patients have developed IBS following GIT infections. These patients have been thought to have peripheral sensitization because of the infection. This temporary peripheral neuroplasticity may persist beyond the period of GIT infection. Such changes to the afferent nervous system may increase the response to visceral stimulation and alter the release of neurotransmitters prompting neurogenic inflammation under circumstances where there ought not to be the release of neurotransmitters [2,3,30,35].

Central Nervous System Changes that Alter Pain in IBS and Depression

Under normal circumstances, the central nervous system can modulate pain sensations from the periphery; these pain pathways interact with and are influenced by stress and emotion [3]. There is evidence from neuroimaging studies that support altered neural processing in the central nervous systems of patients with IBS [2,3]. For example, it has been noted that there is atrophy of portions of the cingulate gyrus and insular cortices of patients with IBS when compared to healthy individuals [2,3,30,35]. In addition, there are changes to the structures around the medial and ventrolateral prefrontal cortex, thalamus and periaqueductal grey [9,33]. These areas of the brain are involved in peripheral pain pathways and act by stimulating the descending pain inhibitory pathways. The more central areas of the brain such as the anterior cingulate cortex and the prefrontal cortex are involved in the emotional component of pain, namely anxiety and depression. The interconnectivity and relationship of these pain pathways affirm the involvement of depression and peripheral neuroplasticity due to IBS to enhanced visceral pain.

Nonetheless, neuroimaging studies have demonstrated changes to the nervous systems of patients with IBS, though it is unknown whether such changes existed before or are the result of the IBS. Patients with IBS will respond differently to rectal stimulation, and this difference between IBS patients may suggest a disparity among individuals in the processing of pain [2] whether it is due to true hypersensitivity or alteration in central and/or peripheral pain signals [3,33].

Role of Depression in IBS

The symptoms of IBS coincide partially with those of depression and other functional somatic disorders; this overlap is marked in some patients to the extent that they meet the criteria for a diagnosis of the respective mental disorders [35,36].

Patients with IBS score higher than healthy controls when it comes to somatization. Negative affect and/or somatization are known to affect health; emotional arousal is associated with physiological consequences and a tendency toward ill health [2]. With IBS and depression, the cortico-limbic-pontine outflow to the GIT is involved in adjusting the autonomic nervous system, neuroendocrine routes, and visceral pain [2]. Adjustments related to these routes are involved in increased pain perception and are modified by early childhood adversity. The emotional motor pathways involve the serotonergic, noradrenergic and opioidergic descending spinal pathways. The role of serotonin, norepinephrine and opioid pathways within the GIT is highlighted in Table 1.

**Table 1:** Hormones normally associated with the central nervous system. GIT: Gastrointestinal tract

Hormone	Characteristics	GIT effects
Serotonin	Source – enteroendocrine cells (90%)	Modulates intestinal secretion and motility; consolidate mucosal and stretch reflexes
	Neurotransmitter	
	Has paracrine functions	
Norepinephrine	Stress hormone	Modulates mucosal integrity and regulates upper GIT reflexes and gastric motor activity
	Involved in the Vagovagal reflex. Associated with elevated levels of IL-6	
Opioids	Interact with mu (µ) receptors in the mucosa, submucosal plexus, the muscle layer	Mucosal protection and regulates motor function
	Involved in the nitric oxide pathway as a mediator	

These pathways are involved in the gastrocolic reflex, gastric emptying, transit time within the intestines and contractions of the bowel wall. Under the influence of the emotional motor system, viz the cortico-limbic-pontine pathway to the GIT, it is demonstrable that emotions in depression are an integral part of the etiology of IBS instead of being a consequence of IBS [2].

Involvement of the Immune System in IBS and Depression

The presence of gut commensals in all GIT tracts hints to the presence of a chronic inflammatory process. The presence of microbes in the absence of illness indicates an equilibrium between the GIT microbes and the immune system. Biopsies of the colon among patients with IBS are usually normal, however, there is evidence that supports the role of inflammation and immune-mediated pathways in IBS [2,30]. Approximately 30% of IBS cases follow infection; in this subset, immune activation and inflammation are sustained for prolonged periods of time [2,35].

Inflammatory mediators cause visceral hypersensitivity, modification of GIT motility and increased gut secretion; these mediators are derived from GIT mucosal mast cells and lymphocytes [3,35]. In addition to inflammatory mediators, mast cells will release, among other things, histamine, tryptase, and serotonin; these paracrine factors will modulate pain, contractility and mucosal permeability. There is evidence of an increase in the number of mucosal mast cells and lymphocytes [35] in the GIT of IBS patients; furthermore, there is an increase in the number of peripheral mononuclear cells. Contrasting reports relating to the level of pro-inflammatory cytokines in IBS biopsies have been published with some studies favoring upregulation of cytokines and others favoring a decrease in these levels [3]. On the other hand, there is agreement with regards to reduction in anti-inflammatory cytokines such as IL-10 and transforming growth factor beta in IBS colonic and rectal biopsies [3]. The mucosal defense that is upregulated by chemokines such as IL-8 and monocyte chemoattractant protein-1 is decreased in IBS [3].

Alteration in the GIT immune system will alter the permeability of the GIT allowing translocation of mediators of inflammation into open circulation. IL-6, as an example, will circulate and cause stimulation of the HPA axis [30]. Depression and other extra-intestinal comorbidities are associated with abnormal levels of IL-8 and tumor necrosis factor alpha [2]. At the same time, depression on its own alters IL-1 and IL-10 levels, further causing a disequilibrium between pro- and anti-inflammatory markers [37,38] which cause a change from normal health to provoke and/or perpetuate IBS symptoms.

Merging the different systems

The complex interplay of the immune system, GIT microbes, and neural and endocrine factors underlie the interrelation of IBS and depression. It is known that under stressful conditions, healthy individuals can adapt and no consequence befalls the GIT. However, in IBS-prone individuals with depression, the co-stimulation of the immune system and the HPA axis frequently precedes symptom flare-ups. This is most prominent among patients who developed post-infectious IBS [3] around the time of a GIT infection and stressful life events. The effect of stress includes modulation of the immune system with an increase in pro-inflammatory cytokines and inflammatory cells such as natural killer cells. At the same time, CRF-related stimulation of the HPA causes an increase in glucocorticoids which are immuno-modulating, favor alteration in the gut microbe flora, and cause an increase and activation of mast cells and granulocytes in the GIT mucosa [2,3]. This increase and activation of immune cells within the GIT leads to the release of inflammatory markers which enhance gut permeability and the translocation of GIT-derived neuroendocrine factors; this cascade of events sets up a cycle and the relationship that is known as the gut-brain axis.

The association of IBS and mood disorders has long been documented; the associated inflammation that occurs due to alteration in GIT microbes and increased cytokines causes oxidative stress leading to damage in areas of the brain such as the hippocampus and amygdala involved in the etiology of depression and other mood disorders [39]. Simultaneously, cytokines alter glutamate levels that modify neuroplasticity and neurogenesis [39,40] by exerting an excitotoxic effect. Cytokines promote CRF-related dysregulation of the HPA axis and altered glucocorticoid activity leading to a prolonged inflammatory response that further adds to the depression. This route of depression is not a universal route, as only those individuals who are vulnerable to such physiological inflammation could end up with depression under such circumstances.

The alteration in GIT microbes is associated with a change in the release of mast cell-derived serotonin, histamine and other mediators of inflammation. Centrally, the role of the background inflammatory state alters metabolism and transport of neurotransmitters involved in mood regulation; these hormones include glutamate and serotonin. As an example, cytokines alter the activity of the heme-containing oxidative enzyme involved in the rate-limiting step of serotonin production. The modified activity of indoleamine 2,3-dioxygenase leads to a reduction in the production of serotonin, thereby promoting depression [39]. It should be noted that among the evaluated studies, one article estimated that 25% of patients with depression had low levels of serotonin or other neurotransmitters such as norepinephrine and dopamine [41,42].

Implications for treatment

Recognition of the multi-directional relationship between IBS and depression can promote collaborative care of the two disorders. Assessing patients suspected of having IBS for depression, stress levels, pain perception and/or anticipation can be used by all physicians regardless of specialty [36]. This biopsychosocial approach accounts for the multi-directional communication observed in IBS and depression; these qualities can be used as a starting point in the treatment of IBS patients.

Treatment of IBS is currently symptomatic, and some of the medications used include anti-spasmodics, prokinetics and bulk-forming agents. This wide array of treatment choices elaborates the multi-factorial and complex pathophysiology involved with IBS. As far as we are aware, there is no drug, drug combination or treatment plan that is universal for all IBS patients [2,43].

Anti-depressants and other psychotropic drugs are routinely used to treat IBS; SSRIs are more favorable than tricyclic antidepressants (TCAs) due to their better side effect profiles. However, as a class, TCAs have been used for and are more effective than SSRIs at modulating pain, hence their use for treating neuropathic pain [2,44]. SSRIs have a prokinetic effect on the small intestine, and this quality can be recruited to enhance the treatment arsenal of IBS. Psychotropics target the HPA axis and influence the autonomic nervous system; this action can influence the efferent sympathetic and parasympathetic routes to deliver a favorable outcome for IBS patients. Anti-depressants, notably SSRIs, have been found to have an anti-inflammatory effect, as they reduce the formation of pro-inflammatory cytokines such as IL-1 while increasing the anti-inflammatory IL-10. This anti-inflammatory quality of SSRIs has been postulated as one of their benefits in treating depression [44]. Further, serotonin modulates intestinal motility and secretion and consolidates mucosal and stretch reflexes within the GIT. There has been renewed interest in targeting these serotonin GIT receptors, and it does sound plausible to use SSRIs and SNRIs as therapeutic options for IBS patients [33,35].

## Conclusions

Periodic abdominal pain without evidence of structural and/or biochemical changes is the hallmark of IBS; the incidence is higher in women and there is an association with mental disorders. Multiple factors are responsible for inciting and perpetuating IBS, and there is overlap in pathways common to both depression and IBS.

The changes in the microbe characteristics and permeability of the GIT are attributable to immune system dysfunction which is found in both IBS and depression. Currently, there is inadequate knowledge of the microbe species that are altered causing dysfunction of the gut-brain axis, and no probiotic composition has been found to be restorative to the normality of the axis. The impact of the immune system changes brings to light potential novel approaches such as targeting IL-10 as part of the therapeutic armament.

Alterations in the HPA axis mediated by CRF in response to stress, altered cytokines, and immune function underlie the complex multifactorial systems involved in IBS and depression. These and other factors are involved in neuroplasticity; the inability of the patient to exhibit neural flexibility attributable, in part, to these factors causes comorbid depression and IBS. Healthy individuals who can adapt and exhibit adequate neuroplasticity are less likely to suffer from comorbid depression and IBS, as we have found in this review. Targeting neuroplasticity is a way neuropsychiatry and gastroenterology can synergize efforts to provide potential curative and preventive therapeutic options.

In summary, there is an association between IBS and depression. However, this association is multifactorial, and there is a need for more research to further elucidate the association and to propose therapeutic and preventative approaches to IBS and depression.
